# A study on the distribution of interleukin-10 gene polymorphisms and their association with febrile seizure susceptibility in children from the Wenzhou region, Zhejiang Province

**DOI:** 10.3389/fpubh.2025.1645018

**Published:** 2025-08-04

**Authors:** Liqiang Lu, Heng Huang, Zhenyao Ding, Ziliang Lin, Xinsheng Lv, Tianjie Gan

**Affiliations:** ^1^Department of Pediatrics, The People’s Hospital of Cangnan, Wenzhou, China; ^2^Division of Neonatology, The People’s Hospital of Cangnan, Wenzhou, China

**Keywords:** febrile seizures, interleukin-10, gene polymorphism, cytokines, pediatric neurology, genetic susceptibility

## Abstract

**Background:**

Febrile seizures (FS) were the most common seizure disorder in young children, with a notable prevalence in the Zhejiang Province region of China. The pathogenesis of FS involves both genetic and environmental factors, particularly the role of cytokines like Interleukin-10 (IL-10), an anti-inflammatory agent. This study examines the distribution of IL-10 gene polymorphisms and their association with FS susceptibility in children from Wenzhou, Zhejiang Province.

**Methods:**

This retrospective study included 77 pediatric patients with FS and a control group of 71 healthy children. The FS group was divided into simple FS (SFS) and complex FS (CFS) subgroups. IL-10 gene polymorphisms at loci 1,082 (rs1800896 G/A), 819 (rs1800871 C/T), and 592 (rs1800872 A/C) were analyzed. IL-10 expression levels were measured, and association with FS susceptibility was evaluated using statistical methods including logistic regression.

**Results:**

IL-10 expression levels were significantly reduced in children with FS (mean: 3.42 ± 1.27 pg./mL) compared to controls (mean: 3.87 ± 1.16 pg./mL; *p* = 0.027). The 1082 (rs1800896) AA genotype was more prevalent in the FS group (44.16%) versus controls (26.76%; *p* = 0.039). Similarly, the 592 (rs1800872) AA genotype was more frequent in the FS group (33.77%) than in controls (14.08%; *p* = 0.014). In CFS cases, the AA genotype of rs1800896 was significantly prevalent (58.33% compared to 31.71% in the SFS group; *p* = 0.044). A strong negative correlation was found between IL-10 levels and FS risk (rho = −0.175, *p* = 0.034), supporting findings from logistic regression that showed higher IL-10 levels were protective (OR = 0.525, *p* = 0.018).

**Conclusion:**

Reduced IL-10 levels and specific IL-10 gene polymorphisms, particularly rs1800896 and rs1800872, were associated with increased susceptibility to febrile seizures in children from Wenzhou. These findings may inform early diagnostic markers, risk stratification tools, and potentially guide personalized treatment strategies for children at higher risk of FS.

## 1 Introduction

Febrile seizures (FS) represent the most prevalent type of seizure disorder in infants and young children, affecting approximately 2–5% of all children under the age of five globally (1). They occur in association with a febrile illness that was not directly related to a central nervous system infection or any acute electrolyte imbalance (2, 3). In particular, the incidence of FS was notably significant in the Chinese pediatric population, with a higher prevalence observed in specific regions such as Wenzhou, Zhejiang Province, underscoring region-specific genetic and environmental influences (4). Although generally benign, FS can cause considerable anxiety for parents and potentially increase the risk of further epilepsy-related disorders later in life, especially in cases of complex febrile seizures (CFS), which were characterized by focal, prolonged, or recurrent episodes. Electroencephalogram (EEG) findings in SFS typically show no abnormalities, whereas CFS may present with interictal epileptiform discharges or other abnormal patterns (5).

The pathophysiology of FS was complex and multifactorial, involving an interplay between genetic susceptibility and environmental triggers such as viral infections and associated fever (6). Recent advancements in genetic research have indicated that cytokines, small proteins that were pivotal in cell signaling, play a significant role in the body’s immune response to infections and were intimately linked to FS (7). In this respect, the cytokine Interleukin-10 (IL-10) has garnered considerable attention due to its prominent anti-inflammatory properties. IL-10 serves as an essential immunoregulatory cytokine, mitigating excessive inflammatory responses by suppressing the expression of pro-inflammatory cytokines and thus potentially modulating excitability within the central nervous system during febrile episodes (8, 9). The rationale for focusing on IL-10 is supported by evidence that its production is significantly elevated in FS, its genetic variant shows distinct distribution between patients and controls, and it exhibits high predictive value for complex FS during viral epidemics, underscoring its critical role in FS pathogenesis and clinical stratification (8, 10, 11).

Previous literature has illustrated the association between altered IL-10 expression levels and susceptibility to various inflammatory disorders, suggesting that decreased levels of IL-10 may result in an inadequate control of inflammation, which was a notable precursor of FS (12–14). Despite these insights, the specific genetic factors influencing IL-10 expression, particularly IL-10 gene polymorphisms, remain insufficiently characterized within the context of FS susceptibility among children, particularly within the diverse genetic backgrounds present in China.

This retrospective study was conducted with the objective of examining the distribution of IL-10 gene polymorphisms and their association with susceptibility to FS in children from Wenzhou, Zhejiang Province. By identifying these association, we aim to better understand the genetic basis of FS and potentially contribute to improved risk assessment and personalized treatment strategies for affected children.

## 2 Materials and methods

### 2.1 Study design

We conducted a retrospective analysis of 77 pediatric patients with FS, admitted to our hospital in Wenzhou, Zhejiang Province, from January, 2024, to December, 2024. The patients were categorized into a febrile seizure (FS) cohort (*n* = 77) and further subdivided based on distinct clinical attributes into a simple febrile seizure (SFS) subgroup (*n* = 41) and a CFS subgroup (*n* = 36). Sample size was determined using the Events Per Variable (EPV) criterion for logistic regression. No fewer than 30 patients were included in each subgroup.

For comparison, 71 healthy children, who attended our outpatient clinic for routine health examinations during the same period, were recruited as the control group. Baseline data for all participants were extracted from the hospital’s medical records. The criteria for classifying SFS included generalized seizures lasting less than 15 min and occurring as a single episode. In contrast, CFS were characterized by either partial seizures lasting between 15 to 30 min, multiple seizure episodes, or fulfilling at least one of these conditions (15).

### 2.2 Inclusion and exclusion criteria

Inclusion Criteria: Participants met the following conditions: (1) Age between 6 months and 5 years; (2) Availability of complete clinical data; (3) Initial diagnosis of FS in the FS group, adhering to the diagnostic criteria: ① Seizures occur during fever (defined as a rectal temperature of ≥38.5°C or an ear temperature of ≥38°C), potentially occurring once or multiple times; ② An EEG performed during the seizure was normal, and head computed tomography (CT) or magnetic resonance imaging (MRI) shows no evidence of central nervous system infection or other seizure-causing conditions; ③ No prior history of FS; ④ In some instances, children may not exhibit fever prior to a seizure but develop it immediately after or during the episode; ⑤ Seizures occur within 3 days of fever onset (16).

Exclusion Criteria: Participants were excluded based on the following conditions: (1) History of previous convulsions; (2) First seizure episode in the FS group occurring at an age outside the 6 months to 5 years range; (3) Seizures arising from other causes, including notable acidosis and electrolyte imbalances; (4) Absence of fever or ear temperature below 38°C during seizures in the FS group; (5) Interictal EEG indicating epilepsy in the FS group; (6) Abnormalities on head CT or MRI associated with seizures; (7) Evidence of a central nervous system infection; (8) History of head trauma, toxic encephalopathy, perinatal brain injury, etc.; (9) Organ dysfunction affecting the heart, lungs, liver, kidneys, etc.; (10) Presence of additional chronic diseases; (11) Inability to detect alleles during genetic testing.

### 2.3 Detection methods

Venous blood samples (5 mL) were collected from the antecubital veins of fasting participants. Within 2 h, these samples were centrifuged using a Beckman Coulter centrifuge (Miami, FL, United States) at 3000 rpm for 10 min. The supernatant, along with the layer containing nucleated cells, was carefully transferred into new centrifuge tubes. The supernatant was stored in liquid nitrogen for the analysis of IL-10 levels, while the nucleated cells were used for genomic DNA extraction.

IL-10 levels were measured using a commercially available ELISA kit (R&D Systems, Minneapolis, MN, United States; Catalog Number: D1000B) according to the manufacturer’s instructions. The absorbance was read at 450 nm using a microplate reader, and concentrations were calculated based on a standard curve.

Genomic DNA extraction was performed according to the protocol provided by the Blood Genomic DNA Extraction Kit [BGI Genomics Co., Ltd., Shenzhen; approval number: Yue Shen Yao Jian Xie (Zhun) Zi No. 2013–1,400,130]. Specifically, 200 μL of proteinase K solution was added to the centrifuge tube based on the sample volume, accompanied by the blood sample and 2 mL of buffer solution GE. The mixture was vortexed for 1 min and incubated at 65°C for 5 mins. Subsequently, the sample was mixed with 2 mL of anhydrous ethanol and transferred to an adsorption column. Following the addition of 2 mL of buffer solution, the mixture was centrifuged at 4000 rpm for 1 min. The process was repeated with additional buffer solution, and after adding 200 μL of elution buffer to the column, genomic DNA was obtained. The purity and concentration of extracted genomic DNA were assessed using a spectrophotometer. The A260/A280 ratio was used to determine purity (target range: 1.8–2.0), and DNA concentration was quantified by measuring absorbance at 260 nm.

The IL-10 gene polymorphism regions were amplified using a PCR instrument (Thermo Fisher Scientific, Waltham, MA, United States) (17). Three loci (1,082, 819, and 592) were screened. For locus 819, the forward primer sequence was 5’-TCATTCTATGTGGTGGACATG-3′, and the reverse primer sequence was 5’-TGGGCCAAGTGGGTAAGAGT-3′, with an enzyme digestion fragment length of 209 bp and the restriction enzyme MsII. For locus 1,082, the forward primer sequence was 5’-ACTACTAAGGCTTCTTTGGGAA-3′, and the reverse primer sequence was 5’-CTACTAAGGCTTCTTTGGGAG-3′, with an enzyme digestion fragment length of 258 bp and the restriction enzyme MnlI. For locus 592, the forward primer sequence was 5’-CCTAGGTCACAGTGACGTGG-3′, and the reverse primer sequence was 5’-GGTGAGCACTACCTGACTAGC-3′, with an enzyme digestion fragment length of 412 bp and the restriction enzyme RsaI. The PCR reaction conditions included an initial denaturation at 94°C for 5 min, followed by 30 cycles of denaturation at 94°C for 45 s, annealing at 55°C for 30 s, and extension at 72°C for 45 s. The resulting fragments were analyzed by 2% agarose gel electrophoresis, stained with ethidium bromide, to determine the three polymorphic sites (18, 19).

### 2.4 Statistical analysis

Statistical analyses were conducted using SPSS version 29.0 software. Continuous data were expressed as mean ± standard deviation (x̅±s), and comparisons between groups were made using independent samples t-tests. Categorical data were presented as percentages (%), with group comparisons conducted using the χ^2^ test. A *p*-value of less than 0.05 was considered statistically significant. For non-parametric or categorical data, Spearman rank correlation analysis was performed. We further explored factors associated with susceptibility to FS in children through both univariate and multivariate analyses.

## 3 Results

### 3.1 Comparison of data between control group and FS group

#### 3.1.1 Basic data

In a comparative study examining baseline characteristics between the control group (*n* = 71) and the FS group (*n* = 77), no statistically significant differences were observed in gender distribution, age, height, weight, family history of FS, and family history of epilepsy (*p*>0.05; Table 1). However, a significant reduction in IL-10 expression levels was detected in the FS group compared to the control group, with mean values of 3.42 ± 1.27 pg./mL and 3.87 ± 1.16 pg./mL, respectively (*p* = 0.027), indicating a potential association between decreased IL-10 levels and increased susceptibility to FS in the studied pediatric population from the Wenzhou region.

**Table 1:** Comparison of baseline data between the control group and the FS group.

Parameters	Control group (*n* = 71)	FS group (*n* = 77)	t/χ^2^	*p*
Gender [n(%)]			0	0.984
Male	37 (52.11%)	40 (51.95%)		
Female	34 (47.89%)	37 (48.05%)		
Age (months)	42.24 ± 1.67	42.39 ± 1.62	0.564	0.574
Height (cm)	90.85 ± 13.92	92.52 ± 12.14	0.778	0.438
Weight (kg)	12.16 ± 3.48	12.87 ± 3.59	1.216	0.226
Family History of Febrile Seizures [n(%)]	2 (2.82%)	7 (9.09%)	1.566	0.211
Family History of Epilepsy [n(%)]	3 (4.23%)	9 (11.69%)	2.761	0.097
IL-10 expression level (pg/ml)	3.87 ± 1.16	3.42 ± 1.27	2.24	0.027

FS, Febrile Seizure; IL-10, Interleukin-10.

#### 3.1.2 Gene frequencies

For the 1,082 (rs1800896 G/A) polymorphism, a significant difference was observed (*p* = 0.039), with the GG genotype being less prevalent in the FS group (11.69%) compared to the control group (23.94%; Table 2). Conversely, the AA genotype was more common in the FS group (44.16%) than in the control group (26.76%). No significant differences were found for the 819 (rs1800871 C/T) polymorphism genotypes (*p* = 0.983), with similar distributions of CC, CT, and TT genotypes across both groups. For the 592 (rs1800872 A/C) polymorphism, a significant difference was found (*p* = 0.014), with a higher frequency of the AA genotype in the FS group (33.77%) compared to the control group (14.08%), suggesting a possible genetic predisposition associated with these IL-10 polymorphisms in the studied population.

**Table 2:** Comparison of gene frequencies between the control group and the FS group [n(%)].

Genotype	Control group (*n* = 71)	FS group (*n* = 77)	χ^2^	*p*
1,082 (rs1800896 G/A)			6.489	0.039
GG	17 (23.94%)	9 (11.69%)		
GA	35 (49.3%)	34 (44.16%)		
AA	19 (26.76%)	34 (44.16%)		
819 (rs1800871 C/T)			0.033	0.983
CC	31 (43.66%)	34 (44.16%)		
CT	24 (33.8%)	25 (32.47%)		
TT	16 (22.54%)	18 (23.38%)		
592 (rs1800872 A/C)			8.486	0.014
AA	10 (14.08%)	26 (33.77%)		
CA	31 (43.66%)	30 (38.96%)		
CC	30 (42.25%)	21 (27.27%)		

FS, Febrile Seizure.

#### 3.1.3 Haplotype frequencies

**Figure 1:**
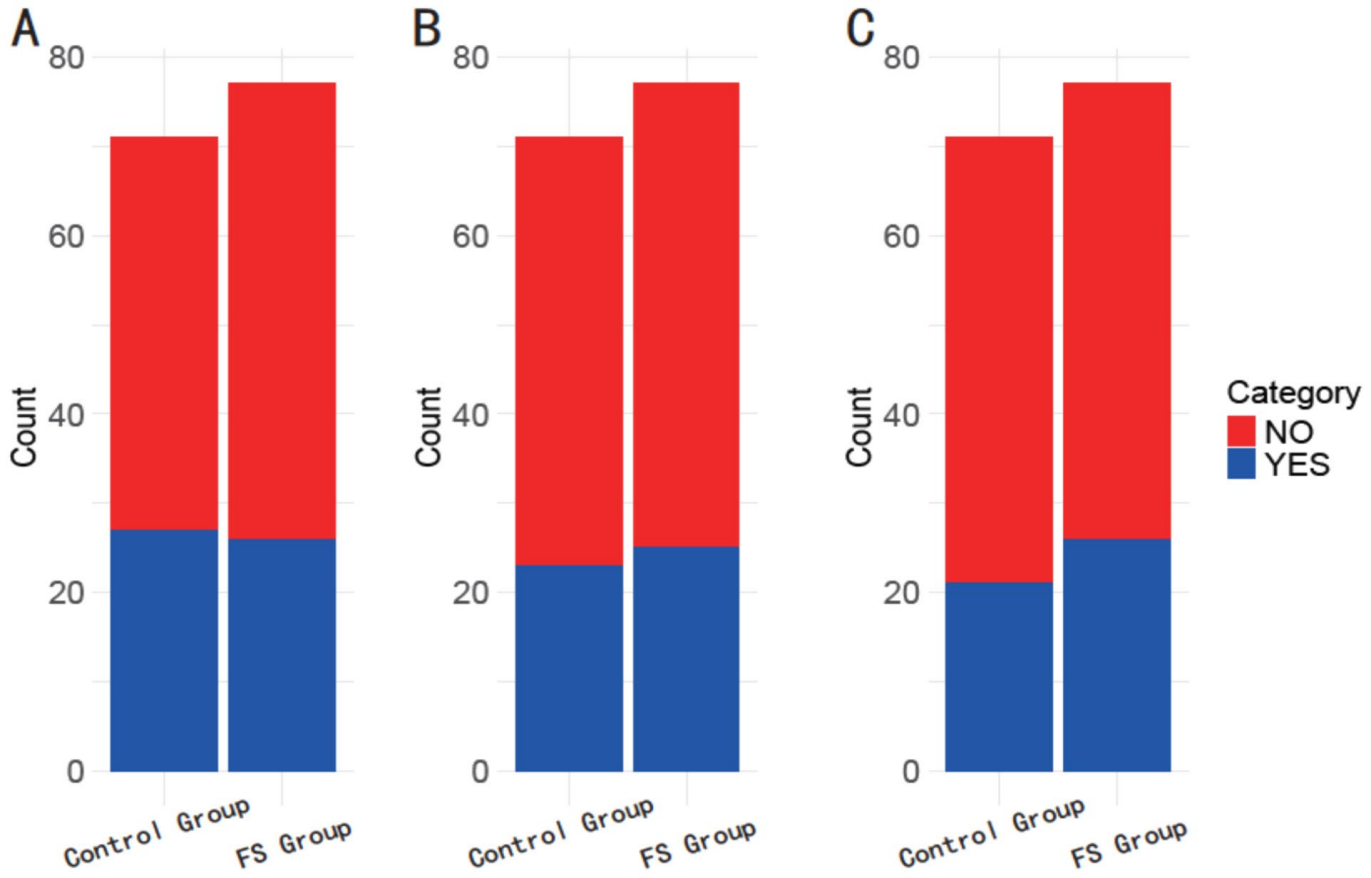
Comparison of haplotype frequencies between the control group and the FS group. **(A)** 1082 (rs1800896) GCC; **(B)** 819 (rs1800871) ACC; **(C)** 592 (rs1800872) ATA.

The GCC haplotype at the 1082 (rs1800896) locus was present in 38.03% of the control group and 33.77% of the FS group (*p* = 0.589; Figure 1). Similarly, the ACC haplotype at the 819 (rs1800871) locus was found in 32.39% of the control group and 32.47% of the FS group (*p* = 0.992). Additionally, the ATA haplotype at the 592 (rs1800872) locus was observed in 29.58% of the control group compared to 33.77% of the FS group (*p* = 0.584). These results suggest that haplotype distributions in these loci do not significantly differ between the control and FS groups in the studied population.

#### 3.1.4 Correlation analysis

A negative correlation was found between IL-10 expression levels and febrile seizure susceptibility (rho = −0.175, *p* = 0.034), indicating that lower IL-10 levels may be associated with increased risk (Table 3). Additionally, the 1,082 (rs1800896 G/A) polymorphism showed a positive correlation (rho = 0.209, *p* = 0.011), suggesting this genotype might contribute to higher susceptibility. Conversely, the 592 (rs1800872 A/C) polymorphism exhibited a negative correlation (rho = −0.224, *p* = 0.006), implying a protective effect against FS. These findings underscore the potential influence of IL-10 gene polymorphisms and expression levels on the risk of FS in the studied pediatric population.

**Table 3:** Correlation analysis of various factors with the susceptibility to FS in children.

Parameters	rho	*p*
IL-10 expression level (pg/ml)	−0.175	0.034
1,082 (rs1800896 G/A)	0.209	0.011
592 (rs1800872 A/C)	−0.224	0.006

#### 3.1.5 Logistic regression analysis

The logistic regression analysis highlighted independent risk factors associated with the susceptibility to FS in children (Table 4). In the multivariate analysis, IL-10 expression levels showed a significant inverse relationship with febrile seizure risk (Coefficient = −0.644, OR = 0.525, *p* = 0.018), suggesting that higher IL-10 levels may reduce susceptibility. Although the 1,082 (rs1800896 G/A) polymorphism was significant in univariate analysis (Coefficient = 0.609, OR = 1.839, *p* = 0.012), it lost significance in the multivariate model (Coefficient = 0.038, OR = 1.039, *p* = 0.990), indicating that its effect might be mediated through other factors. Similarly, the 592 (rs1800872 A/C) polymorphism showed significance in univariate analysis (Coefficient = −0.62, OR = 0.538, *p* = 0.006) but not in multivariate analysis (Coefficient = −0.147, OR = 0.863, *p* = 0.990). These results emphasize the independent protective role of IL-10 expression levels against FS, while the genetic polymorphisms may interact with other variables influencing susceptibility.

**Table 4:** Logistic regression analysis of various factors with the susceptibility to FS in children.

Parameters	Univariate			Multivariate		
	Coefficient	OR	*p*	Coefficient	OR	*p*
IL-10 expression level (pg/ml)	−0.305	0.737	0.029	−0.644	0.525	0.018
1,082 (rs1800896 G/A)	0.609	1.839	0.012	0.038	1.039	0.990
592 (rs1800872 A/C)	−0.62	0.538	0.006	−0.147	0.863	0.990

### 3.2 Comparison of data between SFS group and CFS group

#### 3.2.1 Basic data

In the comparison between the SFS group (*n* = 41) and the CFS group (*n* = 36), there were no statistically significant differences in gender distribution, age, height, weight, family history of FS, family history of epilepsy, age at febrile seizure onset, duration of illness, attack duration, fever temperature, or source of fever (*p*>0.05; Table 5). However, a significant difference was observed in IL-10 expression levels, with the SFS group exhibiting higher levels (3.68 ± 1.22 pg./mL) compared to the CFS group (3.12 ± 1.03 pg./mL; *p* = 0.034). This suggests that increased IL-10 expression may be associated with the presentation of SFS as opposed to CFS.

**Table 5:** Comparison of baseline data between the SFS group and the CFS group.

Parameters	SFS group (*n* = 41)	CFS group (*n* = 36)	t/χ^2^	*p*
Gender [n(%)]			0.103	0.749
Male	22 (53.66%)	18 (50%)		
Female	19 (46.34%)	18 (50%)		
Age (months)	43.18 ± 1.49	42.57 ± 1.61	1.731	0.088
Height (cm)	91.76 ± 11.68	92.49 ± 11.54	0.276	0.783
Weight (kg)	12.63 ± 3.57	12.92 ± 3.41	0.361	0.719
Family History of Febrile Seizures [n(%)]	3 (7.32%)	4 (11.11%)	0.033	0.857
Family History of Epilepsy [n(%)]	5 (12.2%)	4 (11.11%)	0	1
Age at febrile seizure onset(months)	20.53 ± 1.68	19.84 ± 1.57	1.861	0.067
Duration of Illness (h)	4.09 ± 1.38	4.17 ± 1.3	0.257	0.798
Attack Duration (mins)	13.98 ± 1.29	14.51 ± 2.52	1.136	0.261
Fever Temperature (°C)	38.7 ± 0.13	38.69 ± 0.18	0.315	0.754
Source of fever [n(%)]			0.035	0.998
Upper respiratory tract infections	25 (60.98%)	22 (61.11%)		
Lower respiratory tract infections	6 (14.63%)	5 (13.89%)		
Acute gastroenteritis	7 (17.07%)	6 (16.67%)		
Other infections	3 (7.32%)	3 (8.33%)		
IL-10 expression level (pg/ml)	3.68 ± 1.22	3.12 ± 1.03	2.164	0.034

SFS, Simple Febrile Seizure; CFS, Complex Febrile Seizure.

#### 3.2.2 Gene frequencies

For the 1,082 (rs1800896 G/A) polymorphism, there was a statistically significant difference in genotype distribution (*p* = 0.044), with the AA genotype being more prevalent in the CFS group (58.33%) compared to the SFS group (31.71%; Table 6). Conversely, the GG genotype was more common in the SFS group (17.07%) than in the CFS group (5.56%). In contrast, no significant differences were observed in the 819 (rs1800871 C/T) polymorphism (*p* = 0.973), where the distributions of CC, CT, and TT genotypes were similar across both groups. For the 592 (rs1800872 A/C) polymorphism, significant differences were detected (*p* = 0.033), with a higher prevalence of the AA genotype in the CFS group (44.44%) compared to the SFS group (24.39%), and the CC genotype being more frequent in the SFS group (39.02%) versus the CFS group (13.89%). These findings suggest potential genetic factors that may differentiate simple from CFS within the studied population.

**Table 6:** Comparison of gene frequencies between the SFS group and the CFS group [n(%)].

Genotype	SFS group (*n* = 41)	CFS group (*n* = 36)	χ^2^	*p*
1,082 (rs1800896 G/A)			6.244	0.044
GG	7 (17.07%)	2 (5.56%)		
GA	21 (51.22%)	13 (36.11%)		
AA	13 (31.71%)	21 (58.33%)		
819 (rs1800871 C/T)			0.055	0.973
CC	18 (43.90%)	16 (44.44%)		
CT	13 (31.71%)	12 (33.33%)		
TT	10 (24.39%)	8 (22.22%)		
592 (rs1800872 A/C)			6.851	0.033
AA	10 (24.39%)	16 (44.44%)		
CA	15 (36.59%)	15 (41.67%)		
CC	16 (39.02%)	5 (13.89%)		

#### 3.2.3 Haplotype frequencies

The GCC haplotype at the 1082 (rs1800896) locus was observed in 31.71% of the SFS group and in 36.11% of the CFS group (*p* = 0.683; Table 7). Similarly, the ACC haplotype at the 819 (rs1800871) locus had a frequency of 29.27% in the SFS group and 36.11% in the CFS group (*p* = 0.522). The ATA haplotype at the 592 (rs1800872) locus was present in 34.15% of the SFS group and 33.33% of the CFS group (*p* = 0.94). These findings indicate that haplotype distributions were comparable between children with simple and CFS, suggesting that these specific haplotypes may not play a differentiating role in the susceptibility to the type of febrile seizure. 3.2.4 Correlation Analysis.

**Table 7:** Comparison of haplotype frequencies between the SFS group and the CFS group [n(%)].

Haplotype	SFS group (*n* = 41)	CFS group (*n* = 36)	χ^2^	*p*
1,082 (rs1800896)				
GCC	13 (31.71%)	13 (36.11%)	0.166	0.683
819 (rs1800871)				
ACC	12 (29.27%)	13 (36.11%)	0.409	0.522
592 (rs1800872)				
ATA	14 (34.15%)	12 (33.33%)	0.006	0.94

**Figure 2:**
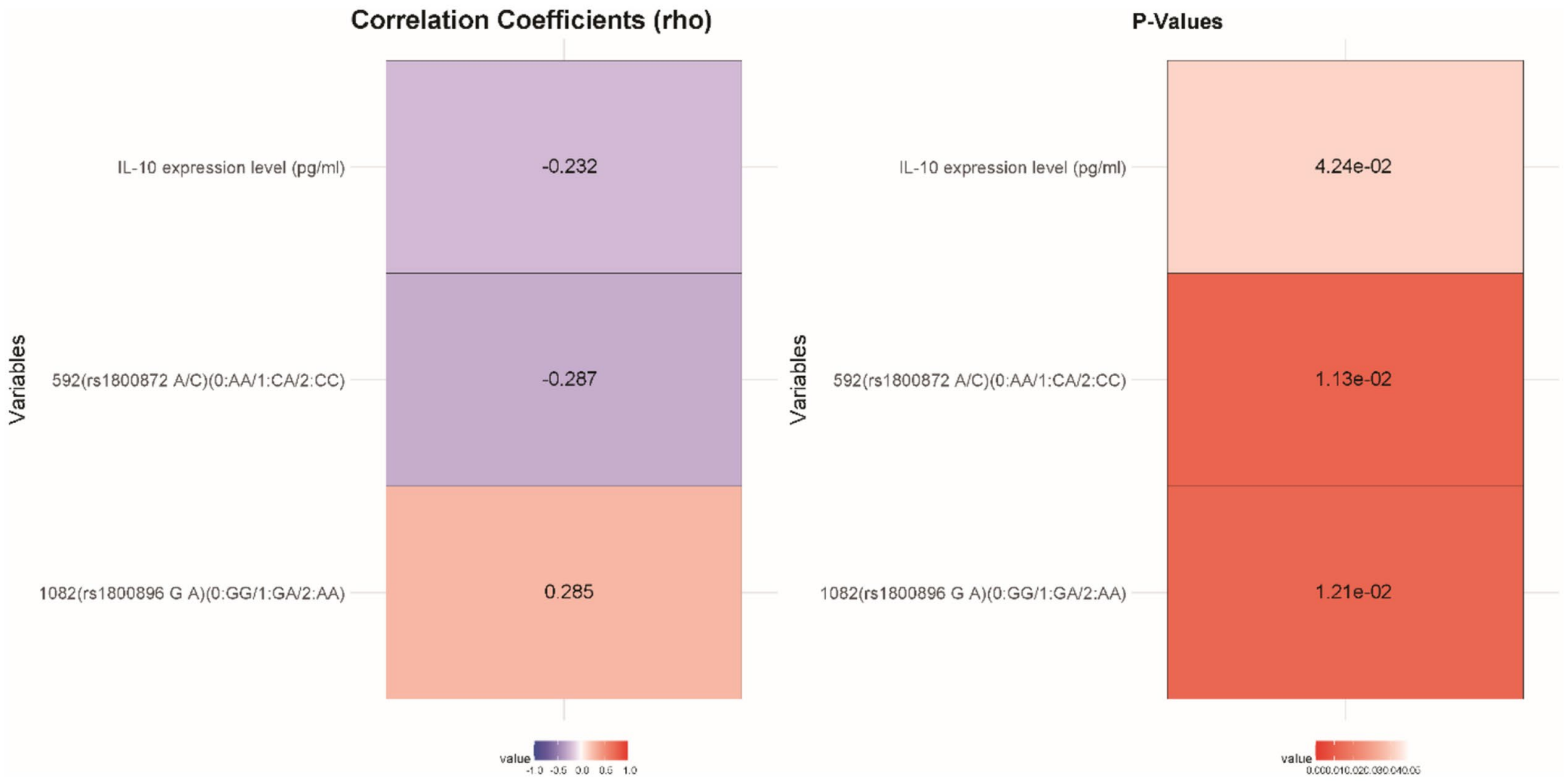
Correlation analysis of various factors with the susceptibility to CFS.

IL-10 expression levels were inversely correlated with CFS susceptibility (rho = −0.232, *p* = 0.042), suggesting that lower IL-10 levels may be linked to a heightened risk of developing CFS (Figure 2). Additionally, there was a positive correlation between the 1,082 (rs1800896 G/A) polymorphism and CFS susceptibility (rho = 0.285, *p* = 0.012), indicating that this genotype may increase susceptibility. Conversely, the 592 (rs1800872 A/C) polymorphism showed a significant inverse correlation (rho = −0.287, *p* = 0.011), implying a protective effect against CFS. These results highlight the potential role of IL-10 expression and specific genetic polymorphisms in influencing the risk of different Types of FS in the studied cohort.

#### 3.2.4 Logistic regression analysis

IL-10 expression levels showed a strong inverse relationship with CFS susceptibility in both univariate (Coefficient = −0.442, OR = 0.643, *p* = 0.038) and multivariate analyses (Coefficient = −0.599, OR = 0.549, *p* < 0.001), indicating that higher levels of IL-10 were protective against CFS (Table 8). The 1,082 (rs1800896 G/A) polymorphism was found to significantly increase susceptibility to CFS, as reflected in both univariate (Coefficient = 0.905, OR = 2.472, *p* = 0.016) and multivariate analyses (Coefficient = 0.039, OR = 1.040, *p* = 0.024). Conversely, the 592 (rs1800872 A/C) polymorphism was associated with a decreased risk of CFS in both univariate (Coefficient = −0.785, OR = 0.456, *p* = 0.013) and multivariate models (Coefficient = −0.191, OR = 0.826, *p* = 0.001), suggesting a protective role. These findings underscore the impact of IL-10 expression and specific gene polymorphisms in modulating the risk of CFS among children in the Wenzhou region.

**Table 8:** Logistic regression analysis of various factors with the susceptibility to different types of FS in children.

Parameters	Univariate			Multivariate		
	Coefficient	OR	*p*	Coefficient	OR	*p*
IL-10 expression level (pg/ml)	−0.442	0.643	0.038	−0.599	0.549	<0.001
1,082 (rs1800896 G/A)	0.905	2.472	0.016	0.039	1.040	0.024
592 (rs1800872 A/C)	−0.785	0.456	0.013	−0.191	0.826	0.001

## 4 Discussion

In this study, we explored the distribution of IL-10 gene polymorphisms and their correlation with febrile seizure (FS) susceptibility among children in the Wenzhou region of Zhejiang Province.

Our results revealed a reduction in IL-10 expression levels in children with FS compared to the control group, suggesting that decreased levels of this anti-inflammatory cytokine might increase susceptibility to FS. IL-10 was a crucial modulator of the immune response, known for its anti-inflammatory properties (20). It works by dampening the immune system’s potential over-reaction during infections or inflammatory responses, which were known triggers for FS in children (21, 22). The diminished IL-10 expression observed in the FS group may fail to counterbalance the pro-inflammatory cytokines that rise during febrile events, leading to an increased risk of seizure occurrence (23). This aligns with previous studies demonstrating that low IL-10 levels were associated with an increased risk of various inflammatory and autoimmune conditions, possibly due to uncontrolled inflammation leading to central nervous system excitability and seizure onset.

Furthermore, our study highlighted the significance of specific IL-10 gene polymorphisms, namely rs1800896 (1,082 G/A) and rs1800872 (592 A/C), in influencing febrile seizure susceptibility. We observed significant differences in genotype distributions for these polymorphisms between children with FS and the control group. The rs1800896 AA genotype was more prevalent in the FS group, suggesting that this variant may promote susceptibility. In terms of mechanistic implications, the rs1800896 polymorphism was located within the promoter region of the IL-10 gene, which may influence the transcriptional activity of the gene itself (24). It was conceivable that different alleles at this locus lead to variations in IL-10 expression, directly impacting the cytokine environment and the inflammatory response during febrile events. The studies on cytokine genetics support this view, showing that promoter polymorphisms can have substantial effects on gene product levels and consequent physiological states (25, 26).

Conversely, the rs1800872 polymorphism demonstrated a distribution that suggests a protective role against FS, with the AA genotype being less frequent and the CC genotype more prevalent in the control group. The location of rs1800872 within the IL-10 gene sequence suggests that alterations in this region could affect mRNA stability or the affinity of transcription factors, thereby modulating IL-10 expression levels (27–29). Such genetic findings were affirmative, considering that genetic predisposition plays a vital role in FS, with previous research showing that multiple gene variants can influence seizure thresholds and inflammatory responses (30).

Interestingly, our IL-10 polymorphism data did not show significant differences in certain haplotype distributions between FS and control groups. This suggests that while individual single-nucleotide polymorphisms (SNPs) can play crucial roles, broader genetic linkages—haplotypes—do not significantly differentiate FS susceptibility in the tested population. This could imply that the effects of IL-10 on FS might be through specific, isolated genetic loci rather than through combination patterns, which emphasizes the importance of pinpoint genetic modifications over broader haplotypic effects in certain genetic backgrounds (31, 32).

Notably, our logistic regression analysis revealed that the rs1800896 and rs1800872 polymorphisms, which showed significance in univariate analyses, lost statistical relevance in the multivariate model. This discrepancy may arise from the interdependence between these genetic variants and IL-10 expression levels. Since these polymorphisms are located in regulatory regions of the IL-10 gene, their effects on FS susceptibility might be mediated through altered IL-10 production rather than acting as independent risk factors (33). When IL-10 levels are included in the multivariate model, the direct association of the polymorphisms with FS risk is attenuated, highlighting that their influence is likely indirect, via modulation of cytokine expression (34).

In contrast, the lack of significant associations in haplotype analyses—despite individual SNPs showing correlations with FS—suggests that the genetic architecture of FS susceptibility in this population is driven by isolated allelic variants rather than combined haplotypic effects. Haplotypes represent linked sets of polymorphisms, and their non-significance here may indicate that the functional impacts of rs1800896 and rs1800872 are context-dependent on their individual alleles rather than their combinations (35, 36). These observations underscore the complexity of genetic regulation in FS, where individual SNPs exert more pronounced effects than broader haplotypic patterns, and their contributions are intertwined with cytokine expression levels (37).

Moreover, our data pointed to distinct profiles between simple and CFS regarding IL-10 levels and genetic predisposition. Lower IL-10 levels were particularly associated with CFS (*p* < 0.001), likely due to a more intense or prolonged inflammatory response in these cases, which fails to be adequately regulated due to insufficient IL-10. Children experiencing complex seizures might have different underlying inflammatory dynamics compared to those with SFS, reinforcing the critical need for understanding individual genetic susceptibilities to tailor clinical approaches (38, 39).

Our logistic regression analysis confirmed that lower IL-10 expression levels were an independent risk factor for FS in both simple and complex forms, hinting at the tangible potential for IL-10 modulation as a therapeutic target (40). Cytokine therapies or other immunomodulatory strategies that enhance IL-10 production or mimic its effects could potentially form part of future preventive strategies against FS in genetically susceptible pediatric populations. This suggests that the role of IL-10 might differ between SFS and CFS, potentially due to variations in inflammatory responses. Studies have shown that CFS is often characterized by more intense or prolonged inflammation compared to SFS, which could be inadequately regulated by insufficient IL-10 levels (10).

The clinical implications of our findings underscore the importance of considering genetic testing and cytokine profiling as part of a comprehensive risk assessment for FS in pediatric patients, especially in populations with a high incidence of fevers due to infectious causes. Genetic testing for IL-10 polymorphisms could become a part of routine evaluation for children with a history of FS or family history suggestive of seizure disorders. This genetic insight opens up opportunities for personalized medicine approaches that take into account individual genetic makeup and cytokine profiles to forecast febrile seizure risk and possibly guide interventions.

Translating IL-10 modulation into therapy presents several challenges, given the genetic variability and immune complexity involved. Genetic polymorphisms within the IL-10 gene can lead to substantial differences in cytokine expression and function among individuals. Additionally, the immune response during febrile episodes is highly complex, involving multiple signaling pathways and interactions between various cell types (41). Therefore, while targeting IL-10 for therapeutic purposes appears promising, it must be approached with caution. Potential strategies may include enhancing IL-10 production through cytokine therapies or developing small molecules that mimic its anti-inflammatory effects. Future studies should focus on elucidating the precise mechanisms by which IL-10 influences FS susceptibility and outcomes, incorporating both genetic and environmental variables to provide a comprehensive understanding.

The study does have its limitations, which must be acknowledged. The relatively small sample size limits the generalizability of our findings, the statistical power was relatively low and the results need to be interpreted with caution. Larger, multi-center studies across diverse populations were warranted to validate these associations. Additionally, while our study focused on genetic factors, the interplay between genetic predispositions, environmental triggers, and other molecular pathways warrants further exploration to provide a holistic understanding of FS. Another limitation is the exclusive focus on children from the Wenzhou region, which may restrict the generalizability of our findings due to potential regional genetic homogeneity. Future studies should expand to multi-ethnic and geographically diverse populations to validate the associations between IL-10 polymorphisms, expression levels, and febrile seizure susceptibility. Additionally, larger cohort studies incorporating gene–environment interaction analyses could further elucidate the mechanistic roles of IL-10 in febrile seizure pathogenesis, enhancing the translational value of these findings.

## 5 Conclusion

In conclusion, our study reinforces the pivotal role of IL-10, both at the genetic and expression level, in modulating febrile seizure susceptibility among children. These findings enhance our understanding of the pathophysiological mechanisms underlying FS, providing a potential pathway for developing targeted preventive strategies that could mitigate risk and improve outcomes for susceptible children.

## Data Availability

The raw data supporting the conclusions of this article will be made available by the authors, without undue reservation.
